# Recent progress on gene-deleted live-attenuated African swine fever virus vaccines

**DOI:** 10.1038/s41541-024-00845-9

**Published:** 2024-03-13

**Authors:** Hiep L. X. Vu, D. Scott McVey

**Affiliations:** 1 Department of Animal Science, and Nebraska Center for Virology, University of Nebraska-Lincoln, Lincoln, NE USA; 2 School of Veterinary Medicine and Biomedical Sciences, University of Nebraska-Lincoln, Lincoln, NE USA

**Keywords:** Viral infection, Translational research

## Abstract

African Swine Fever (ASF) is a highly lethal viral disease in swine, with mortality rates approaching 100%. The disease has spread to many swine-producing countries, leading to significant economic losses and adversely impacting global food security. Extensive efforts have been directed toward developing effective ASF vaccines. Among the vaccinology approaches tested to date, live-attenuated virus (LAV) vaccines produced by rational deleting virulence genes from virulent African Swine Fever Virus (ASFV) strains have demonstrated promising safety and efficacy in experimental and field conditions. Many gene-deleted LAV vaccine candidates have been generated in recent years. The virulence genes targeted for deletion from the genome of virulent ASFV strains can be categorized into four groups: Genes implicated in viral genome replication and transcription, genes from the multigene family located at both 5′ and 3′ termini, genes participating in mediating hemadsorption and putative cellular attachment factors, and novel genes with no known functions. Some promising LAV vaccine candidates are generated by deleting a single viral virulence gene, whereas others are generated by simultaneously deleting multiple genes. This article summarizes the recent progress in developing and characterizing gene-deleted LAV vaccine candidates.

## Introduction

African swine fever virus (ASFV) is a large double-stranded DNA virus of the genus *Asfarvirus*, within the *Asfarviridae* family. The virus infects all members of the family *Suidae*, including domestic pigs, wild boars (*Sus scrofa ferus*), warthogs (*Phacochoerus aethiopicus*), and bushpigs (*Potamochoerus porcus*)^1^. In addition, ASFV can infect Argasid ticks of the *Ornithodoros* genus, which serves as a competent biological vector^2,3^. Domestic pigs infected with ASFV exhibit various clinical signs, ranging from peracute to chronic, depending on the virulence of the viral strain and the age of the infected pig^4^. Highly virulent ASFV strains cause peracute or acute infection, with the mortality rates approaching 100%^5–7^. Moderately virulent ASFV strains often cause sub-acute or chronic infection, with the mortality rates varying between 30% and 70%^8^.

The ASFV genome has a size range of 170–193 kbp and contains 150–190 open reading frames (ORFs)^9^. Over 100 viral proteins have been identified in ASFV-infected cells^10^ and at least 68 proteins are found in the viral virions, with many of them having no known or attributed functions^11^. ASFV has been categorized into 24 genotypes based on the sequence of its major capsid protein, p72^12^. While all 24 genotypes are circulating in Africa, only genotypes I and II have been identified outside this continent^13^. The highly virulent ASFV strains affecting Georgia and Eastern Europe since 2007 and China and Asia since 2018 belong to genotype II^1^.

Pigs recovering from infection with naturally low or moderate virulent ASFV strains are resistant to reinfection with closely related virulent strains^14,15^. These observations clearly demonstrate that the virus can induce protective immunity in pigs. However, the immunological correlates of protection are not yet fully understood. Both humoral and cellular defense mechanisms seem to be crucial for protection against ASFV.

ASFV-specific antibodies can be detected in infected pigs as early as 7–14 days postinfection (dpi)^16,17^. Passive transfer of antibodies collected from hyper-immunized pigs to naïve pigs resulted in protection against lethal challenge infection with a virulent ASFV strain^18^. A positive correlation between the antibody titers and protection outcomes has been demonstrated in pigs vaccinated with a LAV vaccine candidate and challenged with a virulent parental ASFV strain^19^. Conversely, pigs vaccinated with experimental inactivated ASF vaccines generally fail to develop protective immunity, even though they produce anti-ASFV antibodies^20–22^. This observation suggests that pigs infected with a live attenuated vaccine develop a different antibody profile than those vaccinated with an inactivated ASF vaccine.

ASFV-specific T-cell proliferation responses can be detected in pigs at around 10 dpi with a low virulence ASFV strain and reach a maximal response at around 4 weeks post-infection^23^. Both circulating CD4+ and CD8 + T cells obtained from the blood of ASFV-infected animals can respond to ex vivo stimulation with the viral antigens^24^. These cells display characteristics of cytotoxic T lymphocytes (CTLs), as they express cytotoxic granule perforin and can kill infected target cells^23^. These CD8 + T cells play a critical role in protective immunity to ASFV as depletion of CD8 + T cells in immunized pigs with an anti-CD8 monoclonal antibody abrogated the protection^25^.

Various vaccine platforms have been investigated, including inactivated virus vaccines, LAV vaccines, and subunit vaccines. Inactivated virus vaccines do not confer protection, even against a homologous virus strain^20^. Notably, pigs vaccinated with an inactivated ASFV vaccine produce antibodies that are detectable by a commercial ELISA^20^. The failure of inactivated ASFV vaccines to induce protective immunity may be due to factors such as low antigen load, destruction of epitopes during inactivation and downstream processing, ineffective adjuvant formulations, or the absence of non-structural viral protein antigens.

Many experimental subunit vaccines have been developed and tested. These vaccines mainly target the viral structural proteins p32, p54, p72, pp62, and CD2v (as reviewed by^26^). These target proteins are either expressed and purified to be used as protein-based vaccines, or their genes are cloned into DNA expression plasmids or viral vectors to immunize animals. In general, the subunit vaccines can induce adaptive immune responses in vaccinated pigs, but they often fail to confer complete protection against a lethal challenge with a homologous virulent ASFV strain^26^.

LAV vaccines, on the other hand, often confer solid protection against a lethal challenge with a homologous ASF strain. LAV vaccines can be classified into 3 groups: Group I consists of naturally occurring low-virulent ASFV strains^15,27,28^, Group II consists of ASFV strains that are attenuated by consecutively passaging the virus in cell culture^29^, and Group III includes recombinant ASFV strains generated by targeted removal of virulence genes^30–33^. Although naturally low virulent ASFV strains confer protection against closely related strains, they also cause chronic or persistent infection and adverse side effects such as skin lesions and joint swelling in vaccinated pigs^15,28^. Virulent ASFV strains that are serially passaged in non-natural host cell lines (such as Vero cells) become attenuated when tested in pigs, but they lose their ability to confer protection^29^. The most promising approach to generating LAV vaccines for ASFV is targeted deletions of virulence genes from the viral genome, as described in the following sections.

Several gene-deleted LAV vaccine candidates have been generated and studied recently. The virulence genes that are targeted for deletion can be classified into four broad groups: (1) genes that are essential for viral genome replication, (2) genes that belong to the multigene family (MGF) located at both 5′ and 3′ termini, (3) genes that are involved in mediating hemadsorption and putative cellular attachment factors, and (4) novel genes with unknown functions. The complexity of the viral-host interactions in the disease pathogenesis (as observed in infections with other complex DNA viruses) and the complexity of variable host immune responses confound ASFV vaccine discovery and development. This manuscript will focus on these four categories of gene-deleted LAV vaccine candidates.

## LAV vaccine candidates generated by deletion of genes involved in viral genome replication and transcription

The ASFV mainly replicates in macrophages. As non-dividing cells, they have a limited pool of deoxynucleoside triphosphates (dNTPs). Additionally, virus-infected macrophages produce reactive oxygen species (ROS) that induce DNA lesions on the viral genome, causing either miscoded transcription or blockage of DNA and RNA polymerases^9^. Thus, the virus encodes multiple enzymes involved in DNA repair and nucleotide metabolism to increase the dNTP availability as required for viral DNA replication^9^. These gene products are potential targets for the development of LAV candidates.

Recombinant ASFV strains lacking genes encoding thymidine kinase (K196R), an enzyme involved in dNTP synthesis, replicate efficiently in dividing cells such as Vero cells but exhibited a significant attenuation in macrophages^34–38^. Deleting the TK gene from the genome of a primary ASFV isolate partially attenuates the virus, and the resulting recombinant virus maintains the ability to induce protective immunity in pigs. Conversely, deleting the TK gene from the Vero cell-adapted ASFV strain results in over-attenuation and abrogates the virus’s ability to induce immune responses in pigs.

ASFV genomes contain six genes (A859L, F105L, B92L, D1133L, QP509L, and Q706L) that encode for proteins belonging to the RNA helicase superfamily^9^. Some of these viral genes are predicted to have roles in the viral RNA transcription^9^. Deleting the QP509L gene from the highly virulent strain ASFV CN/GS/2018 partially attenuated the virus while co-deletion of QP509L and QP383R completely attenuated the virus. However, the QP509L/QP383R double deletion mutant failed to confer protection^39^. In contrast, deleting A859L from the genome of the highly virulent ASFV isolate, ASFV-G, did not affect virus replication in primary swine macrophage cultures nor virus virulence in pigs^40^.

The E165R and C962R genes encode for a putative dUTPase and NTPase, respectively^41–44^. Deletion of E165R from the Vero cell-adapted ASFV strain BA71V did not affect the virus replication in Vero, but severely reduced virus replication in primary swine macrophage cultures^41^. Interestingly, deleting E165R or C962R from the virulent ASFV-G did not affect virus replication in primary swine macrophage cultures or virus virulence in pigs^45,42^.

The A104R gene encodes for a putative histone-like protein that localizes at the viral DNA replication sites and plays a critical role in viral DNA replication and gene expression^46^. Deletion of A104R from ASFV-G delayed virus replication in swine macrophage cultures but did not significantly affect virus yield. An ASFV-G mutant lacking the A104R gene was partially attenuated, but it failed to confer protection^47^.

In summary, several ASFV mutants lacking genes involved in viral genome replication or transcription have been generated. These mutants were either over-attenuated and unable to induce protective immunity in pigs or not attenuated at all. Thus far, there are no promising LAV candidates that are generated by deleting one of the genes associated with viral genome replication or transcription.

## LAV vaccine candidates generated by deletion of genes belonging to the multigene families

The ASFV genome carries a group of genes called the multigene family (MGF) located in both left- and right-terminal variable regions of the viral genome^9^. MGF genes are classified into five groups (MGF100, MGF110, MGF300, MGF360, and MGF530) based on their average length^9,48^. The numbers of MGF genes are highly variable among ASFV strains, with naturally low virulent or cell-culture-adapted ASFV strains often having fewer MGF genes than highly virulent strains, indicating that these genes may not be essential for virus replication^49,50^. Early studies suggested that MGF genes could determine ASFV tropism^51,52^. In addition, several MGF genes have been demonstrated to suppress the host innate immunity^53^. Thus, MGFs are potential targets for the rational design of LAV vaccines.

Simultaneous removal of six MGF genes (MGF505-1R, MGF360-12L, MGF360-13L, MGF360-14L, MGF505-2R, and MGF505-3R) from the highly virulent ASFV-G did not significantly affect virus replication in primary macrophage cultures, but the mutant virus was fully attenuated in pigs^31^. Intramuscular vaccination of pigs with a single dose of ASFV-G-ΔMGF provided complete protection against a lethal challenge with the virulent parental virus, with only a few pigs experiencing a transient fever^31^. Additionally, this vaccine candidate has been tested for oral vaccination in wild boars. The vaccine induced immune responses in 50% (4/8) of the vaccinated wild boars, and all responders were completely protected against a subsequent challenge infection^54^.

These six MGF genes were also detected in the highly virulent ASFV strain HLJ/18 isolated in China. The resulting six-gene deleted mutant, HLJ/18-6GD, was fully attenuated and induced complete protection against a lethal challenge with the parental virulent virus^55^. These cumulative data suggest that the simultaneous deletion of six MGF genes (MGF505-1R, MGF360-12L, MGF360-13L, MGF360-14L, MGF505-2R, and MGF505-3R) from genotype II ASFVs leads to full attenuation of the virus while maintaining complete protective efficacy. However, it has been reported that the HLJ/18-6GD vaccine candidate reverted to virulence after six back-passages in pigs, but no genetic data was provided to explain how this reversion occurred^55^. The genetic stability of the ASFV-G-ΔMGF vaccine candidate has also been assessed by five back-passages in pigs^56^. Starting from the third passage, inoculated pigs experienced transient fever, with some reaching maximum temperatures of 42 °C on days five and six post-inoculation. Additionally, these animals exhibited mild to moderate clinical signs, such as reduced appetite and lethargy. Importantly, a virus variant with a significant genomic alteration emerged, featuring an 11,197 bp deletion at the 5′-end and a duplication of 18,592 bp from the 3′-end of the genome. Notably, this virus variant was tracked back to the samples collected in the pigs in the first passage. No changes were observed in the MGF region, where genes had been intentionally deleted from the vaccine candidate.

In a separate series of studies, an ~10 kb genomic fragment was deleted from the left variable region of both genotype I (Benin97/1) and genotype II (Georgia2007/1) ASFV strains^57,58^. This deletion affected 10 MGF genes, including the six MGF genes discussed above and the four additional MGF genes (MGF360-9L, 10 L, 11 L, and MGF505-4R). While the manipulation (removal and interruption) of these 10 MGF genes from Benin97/1 (designated Benin-ΔMGF) did not affect virus replication in swine macrophages, it significantly attenuated the virus virulence in pigs^57^. A two-dose intramuscular immunization of domestic pigs with Benin-ΔMGF resulted in 66% to 100% protection, depending on the immunizing virus dose and route of administration^27,57^. Interestingly, different results were observed when the same set of 10 MGF genes was deleted or interrupted from the genotype II Georgia 2007/1 isolate^58^. The resulting recombinant virus (designated Georgia-ΔMGF) exhibited a significant reduction in replication in swine macrophages. All pigs inoculated with Georgia-ΔMGF survived the inoculation without displaying any significant clinical signs, although some of these pigs had moderate viremia. Only 25% of pigs that received a two-dose intramuscular immunization with Georgia-ΔMGF were protected against a lethal challenge with the parental virulent virus^58^. Thus, the relative degree of protection conferred by Georgia-ΔMGF was significantly lower than Benin-ΔMGF, despite having the same deletions.

The cluster of six MGF genes designated as L7L-L11L is located at the right variable region of the ASFV genome. While L7L and L8L are considered members of the MGF100, the functions of other genes in this cluster are unknown. Deletion of L7L-L11L from the ASFV isolate SY18 did not affect virus replication in primary swine macrophage cultures, but significantly attenuated viral virulence in pigs. Out of 12 pigs inoculated with the deletion mutant, 11 survived the infection and exhibited low viremia. All 11 surviving pigs were fully protected against a lethal challenge with the parental virulent virus^59^.

In addition to large deletions of multiple MGF genes, significant effort has been made to evaluate the contribution of individual MGF genes to viral virulence and protection. The MGF505-7R gene inhibits host type-I interferon induction through various mechanisms^60–62^. Deletion of MGF505-7R from virulent ASFV strains had various impacts on viral replication in primary swine macrophage cultures and viral virulence in pigs^60–62^. One hundred percent of pigs survived an intramuscular inoculation with a low dose (1 or 10 HAD50) of the ASFV-ΔMGF505-7R mutant^61,62^, while 43% (3/7) of pigs inoculated with a higher dose (10^5^ HAD50) of the mutant succumbed to the infection^60^. MGF360-9L inhibits IFN-β signaling through targeted degradation of STAT1 and STAT2^63^. Deletion of MGF360-9L alone did not significantly affect ASFV replication in primary swine macrophage cultures. The MGF360-9L deletion ASFV mutant was partially attenuated in pigs, but the inoculation dose used in this study was very low (1 HAD50)^63^. Dual deletion of two interferon antagonists, MGF360-9L and MGF505-7R, from a highly virulent ASFV strain CN/GS/2018 (genotype II) slightly reduced virus replication in swine macrophages while completely attenuating viral virulence in pigs^64^. The resulting recombinant virus (designated ASFV-Δ9L7R) conferred 83.3% (5/6 pigs) protection against a lethal challenge with the parental virulent virus. Other MGF genes, including MGF110-5L, − 6 L, -9L, MGF360-1L, -13L, -14L, and -16R, do not play a significant role in viral virulence^65–69^.

In summary, numerous studies have investigated and described the effects of deleting MGF genes on ASFV virulence and protection. Outcomes can vary when the same set of MGF genes is deleted from different ASFV strains. Nevertheless, multiple MGF-deletion ASFV vaccine candidates have been developed that are fully attenuated in pigs, while also providing complete protection against their corresponding virulent parental ASFV strains.

## LAV vaccine candidates generated by deletion of genes involved in hemadsorption

Most pathogenic ASFV field isolates induce hemadsorption, a phenomenon where red blood cells adhere to the surface of virus-infected cells. However, several naturally low virulent ASFV isolates do not induce hemadsorption^15,70,71^. This observation has led to a hypothesis that viral genes mediating hemadsorption are major virulence factors. Two genes, EP402R and EP153R, have been identified as being responsible for inducing hemadsorption^72^. EP402R encodes a protein homologous to T-cell adhesion receptor CD2, thus, designated as CD2v^73,74^. EP153R encodes a class II transmembrane protein that contains a C-type lectin domain^75^. Hemadsorption is only observed in cells transiently expressing CD2v, but not C-type lectin^74,75^. Thus, only CD2v is directly involved in mediating hemadsorption. The C-type lectin protein might function as a stabilizer of the interaction between the viral CD2v and its corresponding ligand on the surface of the swine erythrocytes^75^. Neither CD2v (EP402R) nor C-type lectin (EP153R) is essential for virus replication in cell cultures^33,75–78^.

Although CD2v is not required for ASFV to replicate in primary swine macrophage cultures, this gene seems to be essential for the virus to replicate in pigs. Except for ASFV-G, deletion of CD2v from other ASFV strains, such as BA71, Kenya-IX-1033, Malawi Lil-20/1, and Congo-a, significantly reduced viral loads in the blood of inoculated pigs. Viral genomic DNA was not detected in the blood of pigs inoculated with BA71-ΔCD2v, Kenya-IX-1033ΔCD2v, and Congo-a-ΔCD2v, while a 4-log reduction of viremia titer was observed in pigs inoculated with Malawi Lil-20/1-ΔCD2v^33,76,78,79^.

ASFV mutants lacking CD2v exhibit a wide range of virulent phenotypes. The deletion of CD2v alone from the genome of Malawi Lil-20/1, ASFV-G, and HLJ/18 isolates did not significantly alter the viral virulence^55,77,78^. On the other hand, deleting CD2v from the genome of ASFV-Kenya-IX-1033 partially reduced viral virulence, with 100% of pigs surviving the inoculation but presenting with fever and reduced appetite^79^. It is noteworthy that only one of nine inoculated pigs exhibited detectable viral genomic DNA at one single time point post-inoculation. Thus, the residual virulence of the ASFV-Kenya-IX-1033-ΔCD2v does not seem to correlate with the virus replication in pigs. Deleting CD2v from the genome of the ASFV strain BA71, adapted to grow in COS-1 cells, completely attenuated the virus^33^. Notably, the BA71-ΔCD2V mutant virus contains seven point mutations in its genomes after 20 successive passages in the COS-1 cells. Six of these mutations were not mapped to any known ORFs. The remaining mutation resulted in a change from histidine 81 to tyrosine within the D250R gene, which encodes a decapping enzyme. Thus, the attenuation phenotype of the BA71-ΔCD2V mutant was ascribed to the deletion of CD2v.

ASFV mutants lacking CD2v exhibited varying degrees of protective potential in experimentally immunized pigs. Deletion of CD2v from Congo-a, an attenuated genotype I ASFV strain adapted to grow in COS-1 cells, completely abrogated virus-induced protection against lethal challenge with the virulent Congo-v strain^80^. Notably, pigs vaccinated with the Congo-a-ΔCD2v mutant mounted similar titers of ASFV-specific antibodies as those inoculated with the parental Congo-a virus^80^. In contrast, 87.5% (7/8) pigs immunized with the Kenya-IX-1033-ΔCD2v mutant survived a lethal challenge with the parental virus, but they all developed a sustained viremia and clinical signs consistent with chronic ASFV infection^79^. Interestingly, all pigs vaccinated with the BA71-ΔCD2V mutant were protected and did not exhibit any significant clinical signs or viremia at any time post-infection with the parental virulent BA71 strain, as well as with challenge with the heterologous strain, E75 (genotype I, same as BA71)^33^. Strikingly, all pigs vaccinated with BA71-ΔCD2v survived a lethal challenge with Georgia 2007/1, a highly virulent genotype II ASFV strain, without showing any signs of ASF.

## LAV vaccine candidates generated by deletion of genes with no known functions

### NL, UK, and 9GL

Earlier studies identified three highly conserved genes that are essential for viral virulence, namely NL (DP71L), UK (DP96R), and 9GL (B119L)^81,82^. The NL (DP71R) gene encodes for a protein with two isoforms: the long form contains 184 amino acids, and the short form contains 70-72 amino acids^83^. Both forms of this protein carry a domain significantly similar to ICP34.5, a neurovirulence-associated protein of herpes simplex virus^82,83^. Deleting the NL gene from highly virulent ASFV strains, E70 or ASFV-G, significantly reduced their respective virulence, with 80% - 100% of inoculated pigs surviving the infection and presenting only transient febrile responses^82,84^. However, deleting this gene from two other virulent ASFV isolates, Malawi Lil-20/1 or Pretoriuskop/96/4, did not alter the virus virulence^85^.

The UK (DP96R) gene encodes for a 15 kDa protein that is expressed at early times in virus-infected cells. Deletion of DP96R from the pathogenic ASFV strains, E70 or ASFV-G, did not affect the virus replication in primary swine macrophage cultures^81,84^. However, the contribution of the UK gene to viral virulence is strain-dependent. While deleting the UK gene from the ASFV strain E70 significantly reduced its virulence, removing it from ASFV-G did not reduce the viral virulence^84^.

NL and UK were simultaneously deleted from the genome of the naturally attenuated OUR T88/3 strain to further enhance its safety. Although the OUR T88/3 virus can induce protection against a lethal challenge with related virulent viruses, it causes adverse reactions, including fever and joint swelling, to a significant portion of immunized animals^28^. Dual deletion of these two genes did not significantly affect the virus replication in macrophages in vitro but significantly reduced its protective potential^86^.

The 9GL (B119L) gene is a homolog to ERV1, which has a role in oxidative phosphorylation^87^. Deleting the 9GL gene from the genome of the ASFV strain Malawi Lil-20/1 severely impaired virus replication in swine macrophages and completely attenuated the virus. Pigs immunized with this deletion mutant were protected against lethal challenge infection with the parental strain^87^. Subsequently, this gene was also deleted from the genome of the virulent ASFV-G^88^. The resulting ASFV-G-Δ9GL mutant exhibited a significant replication deficiency in swine macrophages but still induced a lethal disease when intramuscularly inoculated to pigs at a dose of 10^4.0^ HAD50. At a lower dose (10^3.0^ HAD50), the ASFV-G-Δ9GL mutant was attenuated and conferred complete protection against lethal challenge with the virulent parental virus^88^. Simultaneous deletion of 9GL (B119L) and UK genes from the virulent ASFV-G virus fully attenuated the virus. All pigs inoculated with the double gene-deleted mutant ASFV-G-Δ9GL/ΔUK survived the infection and exhibited no noticeable clinical ASF signs even when inoculated with a high dose of 10^6.0^ HAD50. Vaccination with the ASFV-G-Δ9GL/ΔUK mutant induced fully protected against a lethal challenge with the parental virus, with protective immunity achieved as early as two weeks post-vaccination^32^. However, when the NL gene was deleted from the ASFV-G-Δ9GL/ΔUK, the resulting triple gene-deleted mutant ASFV-G-Δ9GL/ΔNL/ΔUK appeared to be over-attenuated and failed to elicit protective immunity in vaccinated pigs^84^.

Recently, the 9GL and UK genes were co-deleted from HLJ/18, a highly virulent genotype II ASFV isolate genetically similar to ASFV-G^89^. Like ASFV-G, the dual deletion of 9GL and UK from HLJ/18 fully attenuated the virus when tested in pigs^55^. However, unlike ASFV-G, pigs vaccinated with the double-gene deleted mutant HLJ/18-Δ9GL/UK were not protected against a lethal challenge with the parental virulent virus^55^.

In summary, the 9GL and UK genes are major virulence determinants. Co-deletion of these two genes from virulent genotype II ASFV strains (ASFV-G, or HLJ/18) fully attenuated the viruses. However, the protective efficacy of the resulting double deletion mutants was inconsistent^32,55^.

### I117L

The I177L gene encodes an uncharacterized protein expressed late during the virus replication cycle^30^. I177L is highly conserved among ASFV strains. Deletion of I177L from the highly virulent ASFV-G significantly reduced virus replication in primary swine macrophage cultures and completely attenuated viral virulence in swine. Pigs inoculated intramuscularly with the ASFV-G mutant lacking the I177L gene (designated ASFV-G-ΔI177L) at a dose ranging from 10^2^ to 10^6^ HAD50 remained clinically healthy. A single-dose intramuscular inoculation with 10^2^ HAD50 of ASFV-G-ΔI177L induced complete protection against a lethal challenge with the parental virulent virus. Remarkably, genomic DNA of challenge virus was not detected from tissue samples of ASFV-G-ΔI177L-inoculated pigs following challenge infection, suggesting that this mutant could induce sterilizing immunity^30^. The ASFV-G-ΔI177L virus was found to induce protection in pigs when administered via the oronasal route, although a significantly higher inoculation dose was required compared to intramuscular administration^90^.

The ASFV-G-ΔI177L virus established a systemic infection in pigs, with relatively high titers of infectious virus (up to 10^7.0^ HAD50/mL) detected in the blood of inoculated pigs until the end of a 28-day monitoring period^30,90–92^. Additionally, the virus persisted in inoculated pigs for an extended period of time, with genomic DNA of the vaccine virus detected in the tonsil and spleen of some vaccinated pigs at 49 days post-vaccination, corresponding to 21 days after the pigs were challenged with the parental virus^30^. Under experimental conditions, pigs inoculated via an intramuscular or oronasal route with various doses of ASFV-G-ΔI177L did not shed the virus to sentinel pigs cohabitating for 28 days^30,90,92^. However, under field conditions, 50% (5/10) of the sentinel pigs comingling with ASFV-G-ΔI177L-inoculated pigs developed ASFV-specific antibodies^92^, indicating possible virus transmission.

The ASFV-G-ΔI177L virus remained genetically stable after five consecutive back-passages in pigs^92^. No significant genomic differences were detected between the original stock virus and the virus obtained after the fifth back-passage in pigs. Furthermore, no clinical signs specific to ASF were observed in pigs during the successive back-passages, even though the infectious virus was detected in their blood with increasing titers after each passage. The authors speculated that the rising virus titers observed during back-passages could have been due to the higher titer of the inoculum used^92^.

The ASFV-ΔI177L virus was developed from an ASFV strain propagated in primary swine macrophage cultures. This vaccine candidate was subsequently adapted to PIPEC, a subclone of the porcine fetal kidney cell line stably expressing bovine αVβ6 integrin^93^. After 11 passages in PIPEC, the ASFV-ΔI177L virus acquired significant deletion from the left variable regions of the viral genome. Nevertheless, the resulting virus, designated ASFV-ΔI177L/ΔLVR, maintained the ability to replicate in primary swine macrophage cultures while also being able to replicate and produce high virus titers in PIPEC cells^93^. Moreover, the ASFV-ΔI177L/ΔLVR vaccine strain preserved the attenuation and protective efficacy of its parental virus, ASFV-ΔI177L^93^.

### Other individual virulence genes

The I226R gene is a conserved gene located in the right variable region of the viral genome and is transcribed at the late stage of infection^94^. The protein encoded by the I226R gene localizes to the viral replication apparatus in the cytoplasm, but its specific functions remain unknown. When the I226R gene was deleted from the genome of the virulent ASFV isolate SY18, virus replication in primary swine macrophage cultures was slightly reduced, but viral virulence in pigs was completely attenuated. Pigs inoculated intramuscularly with two different doses of the SY18-ΔI226R mutant (10^4^ and 10^7^ TCID50) were clinically normal, although they all exhibited moderate viremia titers, which persisted to the end of the 21-day observation period. All pigs vaccinated with the SY18-ΔI226R mutant were protected against a lethal challenge with the parental virus^94^.

The DP148R gene is located at the 3′ end of the viral genome and is expressed at the early stages of infection. Deleting DP148R from the genome of the virulent genotype I ASF isolate Benin did not affect virus replication in primary swine macrophage cultures^95^. Pigs immunized intramuscularly with 10^3^ HAD50 of the Benin virus lacking DP148R (BeninΔDP148R) exhibited transient fever with moderate virus titers in their blood, but they all survived the inoculation. All pigs that received a two-dose intramuscular immunization with the BeninΔDP148R mutant were protected against a challenge with the parental virulent virus without exhibiting any signs of ASF. The ability of BeninΔDP148R to induce protective immunity was also tested through intranasal immunization. Transient fever was observed in five out of six pigs after the primary dose of intranasal inoculation, but no clinical signs were observed after the booster immunization or the challenge infection with the parental virulent virus. Only one out of six pigs intranasally immunized with BeninΔDP148R was not protected against the challenge infection, presumably because it was not infected with the vaccine virus^95^.

The A137R gene encodes for a late-expressed structural protein with no known functions. Deletion of A137R from ASFV-G slightly reduced virus replication in primary swine macrophage cultures but completely attenuated virus virulence in pigs^96^. After intramuscular inoculation with 10^2^ HAD50 of the mutant, all pigs remained clinically healthy during the 28-day observation period, despite having medium to high viremia titers that persisted until the end of the observation period. A single-dose intramuscular inoculation with the ASFV-G-ΔA137R induced full protection against a lethal challenge with the virulent parental virus, and no evidence of replication of the challenge virus was observed. However, the ASFV-G-ΔA137R virus can establish a persistent infection in vaccinated pigs for an extended time^96^.

The H108R gene encodes for a late-expressed protein with no attributed functions^97^. The A151R gene encodes for a non-structural protein expressed throughout the virus replication cycle^98^. Deletion of either the H108R or A151R gene from the genome of ASFV-G slightly reduced virus replication in swine macrophage cultures. ASFV-G mutants lacking H108R or A151R were partially attenuated, with 80% (4/5) of pigs surviving an intramuscular inoculation with 10^2^ HAD50 of the virus, and the surviving pigs were protected against a subsequent challenge with the parental virulent virus^97^.

## LAV vaccine candidates generated by simultaneous deletion of multiple virulence determinants

The gene encoding CD2v protein (EP402R) is often co-deleted with other viral virulence genes to enhance the safety profile of LAV vaccine candidates. In most cases, simultaneous deletion of CD2v with other viral genes did not significantly affect virus replication in primary swine macrophage cultures but dramatically reduced or diminished the presence of infectious virus in the blood of immunized animals. Co-deletion of CD2v with other virulence genes resulted in a wide range of effects on the protective potential of the LAV vaccine candidates.

Co-deletion of CD2v and DP148R was conducted on Benin, a virulent genotype I ASFV strain. The mutant carrying a single deletion of DP148R (Benin-ΔDP148R) was attenuated but persisted in the blood of vaccinated pigs for an extended period^95^. Additional deletion of CD2v from Benin-ΔDP148R significantly shortened the duration of viremia in immunized pigs without affecting the degree of protection against the lethal challenge with the virulent Benin strain^99^.

A combination of deletions was performed on the virulent ASFV GZ201801 (genotype II), targeting three MGF genes (MGF360-12L, -13L, -14L) together with the C-type lectin protein (encoded by EP153R) and CD2v. The resulting mutant virus replicated efficiently in primary swine macrophage cultures but was completely attenuated in pigs. A single intramuscular immunization of pigs with the mutant virus provided full protection against a lethal challenge with the parental virulent virus^100^.

As described above, the ASFV-G vaccinate candidate lacking 9GL (ASFV-G-Δ9GL) was partially attenuated but could provide complete protection against a lethal challenge with the parental virulent virus^88^. To further improve the safety profile of the ASFV-G-Δ9GL virus, six genes of the MGF360 and MGF505 gene families were deleted from the viral genome. However, this additional deletion resulted in an over-attenuated virus that failed to replicate or induce immune responses in pigs, even when administered at a dose of 10^6^ HAD50^101^. Similarly, when CD2v was co-deleted from the genome of ASFV-G-Δ9GL, its protective potential was lost^102^. It should be noted that the deletion of CD2v alone did not affect the virulence of ASFV-G^77^.

The CD2v and UK genes were simultaneously removed from the virulent ASFV strain SY18 (genotype II). The resulting double gene deletion mutant SY18-ΔCD2v-ΔUK was fully attenuated and did not induce viremia in vaccinated pigs. Interestingly, all pigs immunized by an intramuscular inoculation with the SY18-ΔCD2v-ΔUK mutant survived a lethal challenge infection with the parental virus. No significant clinical signs were observed, and only minimal viral genomic DNA was detected in the blood at later time points (18 and 21 days) after challenge infection^103^.

In another study, a seven-gene deleted ASFV vaccine candidate was generated by simultaneously deleting seven genes, including six MGF genes (MGF505- 1R, -2R, -3R, MGF360-12L, -13L, -14L) together with the CD2v gene from the virulent ASFV isolate HLJ/18^55^. The resulting recombinant virus, HLJ/18-7GD, was fully attenuated when tested in young pigs and pregnant sows. A single-dose intramuscular immunization of young pigs with 10^3^ or 10^5^ TCID50 of HLJ/18-7GD virus conferred protection against lethal challenge infection with the virulent parental virus. All four pigs that received 10^3^ TCID50 of HLJ/18-7GD were febrile, whereas only one of the four that received 10^5^ TCID50 was febrile. This suggests that the HLJ/18-7GD mutant induced protection in a dose-dependent manner. The HLJ/18-7GD mutant replicated minimally in inoculated pigs, as young pigs inoculated intramuscularly with up to 10^7.7^ TCID50 of the HLJ/18-7GD virus did not become viremic. Viral genomic DNA was sporadically detected in lymph nodes up to day 14 post-inoculation, but naïve pigs inoculated with homogenates of these lymph nodes did not become infected. Similarly, viral genomic DNA was not detected in blood or tissue samples of naïve pigs inoculated with the pools of blood samples collected from pigs inoculated with HLJ/18-7GD on days 5 and 10 post-inoculation. The results suggest that the HLJ/18-7GD vaccine candidate is safe and effective.

## Attempts to develop LAV vaccine candidates with a DIVA marker

Differentiating infected from vaccinated animals (DIVA) is a desirable feature of a vaccine as it enables the use of serological tests to detect vaccinated animals that are subsequently infected with the corresponding wild-type viruses^104,105^. Typically, live-attenuated DIVA vaccine strains lack at least one antigenic component, known as a marker antigen, compared to the corresponding wild-type virus^106^. Therefore, a differential serological assay developed based on the marker antigen can be used to detect naturally infected animals within the vaccinated population. Theoretically, all gene-deleted ASFV vaccine candidates can serve as DIVA vaccine strains as they lack at least one viral protein compared to the wild-type ASFV strains. However, the virulence genes deleted from the viral genome to achieve attenuation might not be immunogenic. Thus, differential serological tests based on those proteins might not provide optimal diagnostic capabilities. There are some attempts to delete additional genes encoding for immunogenic proteins from some of the promising LAV vaccine candidates to endow them with the DIVA potential.

In one study, the immunogenic protein encoded by the late-transcribed gene E184L was selected for removal from the genome of a promising LAV vaccine candidate ASFV-G-ΔMGF^107^. Antibodies against E184L-encoded protein are consistently detected in pigs surviving a natural infection with ASFV^108^ or from pigs that were immunized with different experimental LAV vaccine candidates, including ASFV-G-ΔMGF, and ASFV-BA71-ΔCD2^31,33^. Deletion of E184L from the genome of the highly virulent ASFV-G only slightly affected virus replication in primary swine macrophage cultures and partially attenuated viral virulence when tested in pigs. However, when E184L was deleted from the genome of the LAV vaccine candidate ASFV-G-ΔMGF, the resulting virus lost its protective potential completely, mainly due to the inability of the mutant virus to replicate in pigs^107^.

In another study, two immunogenic genes, MGF100-5L and -6L, were identified as potential DIVA marker antigens. Antibodies against MGF100-5L and -6L were consistently detected in pigs vaccinated with different experimental LAV vaccine candidates. Although the MGF100-5L and -6L genes do not play a major role in ASFV virulence, deletion of MGF100-5L and -6L from the genome of the LAV vaccine candidate ASFV-ΔI177L significantly reduced its protective potential^69^.

As described above, the LAV vaccine candidate HLJ/18-7GD was generated by simultaneously deleting seven genes, including six MGF genes (MGF505- 1 R, -2R, -3R, MGF360-12L, -13L, -14L) and CD2v, from the highly virulent ASFV isolate HLJ/18^55^. A dual indirect ELISA based on p54 and CD2v proteins (encoded by E183L and EP402R, respectively) was developed to differentiate serum antibodies from pigs infected with wild-type ASFV or those vaccinated with the HLJ/18-7GD vaccine candidate. Antibodies specific to CD2v were detected in 97.8% of pigs infected with wild-type virus but only 2.8% of those vaccinated with the HLJ/18-7GD. Conversely, antibodies specific to p54 were detected in 100% of pigs infected with wild-type ASF and 96.5% of pigs vaccinated with the HLJ/18-7GD vaccine^109^. The sensitivity of the CD2v-based ELISA reported in this study was relatively high, but it is important to note that there are naturally occurring ASFV strains with low or moderate virulence that do not express CD2v^15,70,71^. Therefore, the CD2v-based ELISA may not be able to detect pigs infected with these natural non-hemagglutinating ASFV strains.

## Cell lines for ASFV propagation

ASFV has a strong tropism for cells of the monocyte/macrophage lineage. Therefore, primary swine macrophage cultures are commonly used for the isolation and propagation of ASFV in vitro. Several continuous or immortalized swine macrophage cell lines^110–112^, as well as a fetal wild boar lung cell line (WSL)^113^, have been demonstrated to be highly susceptible to ASFV infection. Moreover, monkey kidney cell lines such as COS-1 and MA104 can support ASFV replication without requiring an adaptation^114,115^. ASFV grown in WSL or COS-1 cell lines have the same virulence as ASFV grown in primary swine macrophage cultures^33,113^.

ASFV can be adapted to replicate in several continuous cell lines, including monkey kidney cells (such as Vero cells) and human embryonic kidney cells (HEK-293T cells) by successive passages^29,116,117^. ASFV isolates adapted to these cell lines typically have gene deletions within both the left- and right-variable regions of the viral genome^29,116,118,119^. The number of genes deleted from the viral genomes tends to increase with the number of passages performed^29,116^.

ASFV strains adapted to continuous cell lines may exhibit varying degrees of attenuation and protective efficacy, depending on the cell types used and the number of passages performed. For example, the virulent ASFV-G was adapted to Vero cells (designated as ASFV-VP), and its attenuation and protective potential were evaluated. While ASFV-G at passage 30 was fully virulent at a dose of 10^2^ HAD50 in pigs, ASFV-VP at passages 60 and 80 were partially attenuated and lethal only at a dose of 10^4^ HAD50. At passage 110, the virus was completely attenuated and was not lethal even when administrated at the dose of 10^6^ HAD50. Pre-inoculation of pigs with ASFV-VP at passages 60, 80, or 110 did not induce protection against a lethal challenge with the parental virulent virus^29^. Another strain, BA71, also became completely attenuated after adaptation to Vero cells. Like ASFV-G, the BA71 adapted to Vero cells lost the ability to induce protective immunity in pigs^33^.

Most of the gene-deleted ASFV vaccine candidates are generated from ASFV isolates that were initially propagated in primary swine macrophage cultures. There are only a few promising LAV candidates, including BA71-ΔCD2V^33^ and Kenya-IX-1033-ΔCD2v^79^, that were generated using ASFV strains adapted to grow in continuous cell lines. The ASFV-G-ΔI177L was generated from an ASFV isolate propagated in primary swine macrophage cultures but was subsequently adapted to grow in a continuous porcine epithelial cell line called PIPEC. Adapting the ASFV-G-ΔI177L vaccine candidate to PIPEC resulted in a significant additional deletion from the virus genome, but such deletion did not affect the protective potential of this vaccine candidate^93^.

## Conclusions and future directions

The current literature suggests that rational deletion of virulence genes represents the most promising approach to the development of efficacious vaccines against ASFV. Several gene-deleted LAV vaccine candidates have been reported, which exhibited a wide range of safety and efficacy profiles. Viral replication in primary swine macrophage cultures is commonly used to predict viral virulence in pigs. However, several LAV vaccine candidates were fully attenuated when tested in pigs, even though they replicated normally in primary cell macrophage cultures^31,33,57,59^. Therefore, the attenuation phenotype of the resulting gene-deleted ASFV strains is mainly evaluated in young pigs.

The primary route of inoculation is intramuscular, and the common dose of inoculation is 10^2^ HAD50 per pig. In some instances, high inoculation doses of up to 10^6^ HAD50 were used to further evaluate the safety of the vaccine candidates. Quantitative variables used to evaluate attenuation phenotypes include viral load in blood, body temperature, and survival rate after inoculation. Qualitative variables such as lethargy, anorexia, respiratory distress, vomiting, skin hemorrhage, and bloody diarrhea are also reported. LAV vaccine candidates are considered fully attenuated when all pigs survive the inoculations and present with mild, transient, or no fever or other clinical signs.

Most LAV vaccine candidates induce a systemic infection, and infectious virus or viral genomic DNA can be detected in the bloodstream for varying durations. In general, LAV vaccine strains must be able to replicate in pigs to induce protection. However, some LAV vaccine candidates induce minimal or no viremia while still conferring protection against lethal challenge infection^55,79^.

Viral transmission was also evaluated in some studies by co-housing sentinel pigs in the same room as LAV vaccine-inoculated pigs^30,88,93^. Some LAV vaccine candidates were reported not to transmit the detectable virus to commingled pigs under experimental conditions^30^, but they were later found to transmit the virus to sentinel pigs when larger numbers of pigs were included and tested under field conditions^92^.

Reversion to virulence is a major concern of the LAV ASF vaccines. However, the potential for reversion to virulence has been evaluated in only a few LAV vaccine candidates. One LAV vaccine candidate (HLJ/18-6GD) was reported to revert to virulence, while the two other vaccine candidates (ASFV-G-ΔI177L and HLJ/18-7GD) were reported to be stable after five or six back-passages in pigs^55,92^.

The protective efficacy is commonly evaluated by challenging vaccinated pigs with the virulent parental ASFV strains via intramuscular inoculation at 3-4 weeks post-immunization. The common virus dose used for challenge infection is 10^2^ HAD50, which often results in 100% mortality in non-vaccinated control pigs. In most cases, all immunized pigs survived a lethal challenge with the corresponding virulent parental strain, although they might exhibit low to moderate viremia titers and transient fever following the challenge infection. Remarkably, some studies reported sterilizing immunity where immunized pigs were protected against infection with the virulent parental virus and exhibited no fever or any other clinical signs^30,33,93^.

While most studies have been focused on evaluating protection against homologous strains, there are a few studies that report heterologous protection against different genotypes. For example, the BAV71-ΔCD2v vaccine candidate (genotype I) was reported to confer full protection against ASFV-Georgia 07 (genotype II)^33^ and partial protection against an infected tick challenge using *Ornithodoros sp*. soft ticks naturally infected with RSA/11/2017 strain (genotype XIX)^120^. Although several LAV vaccine candidates based on ASFV-G (genotype II) have been reported to confer solid homologous protection, there are no reports regarding their heterologous protection against ASFV of different genotypes.

Contradicting results have been reported when the same set of viral genes is deleted from two different ASFV isolates^32,55,57,58^. Additionally, unpredictable synergistic effects of co-deletion of two sets of viral genes have been reported^77,102^. There are no reliable methods to predict the effects of a viral gene on viral virulence and protective potential. Thus, the attenuation and protective efficacy of gene-deleted ASFV vaccine candidates must be evaluated in pigs.

Although several continuous macrophage cell lines have been developed, most LAV vaccine candidates are still propagated in primary swine macrophage cultures, which might be contaminated with unknown pathogens. Furthermore, the viability and susceptibility of the primary cultures can vary among batches of cell preparations, posing a significant challenge to the large-scale production of the vaccines. Adapting the well-characterized LAV vaccine candidates propagated in primary macrophage cultures to continuous cell lines might result in additional mutations, which may affect the safety and efficacy profiles of the vaccine candidates. From a manufacturing standpoint, it might be better to develop LAV vaccine candidates based on ASFV strains stably adapted to grow in continuous cell lines. Going forward, the stability of cell lines selected for potential manufacture as well as the stability of the seed strain virus constructs through the stages of upstream virus production will be important.

Initial attempts to develop a DIVA marker vaccine by deleting additional immunogenic antigens from the LAV vaccine candidates have been unsuccessful because the additional deletion of immunogenic marker antigen from the genome of potential LAV vaccine strains often abrogates the vaccine efficacy^69,107^. The CD2v-based ELISA has been developed to serologically differentiate pigs vaccinated with the ASFV vaccine lacking CD2v and those infected with wild-type ASFV strains (Wang et al., 2022). However, the CD2v-based ELISA has a major shortcoming in that it would not be able to serologically detect pigs infected with naturally occurring low or moderately virulent ASFV strains that lack CD2v. Therefore, there is a need to identify immunogenic antigens that can be safely removed from the genome of LAV vaccine candidates without affecting virus replication and its ability to induce protective immunity.

In conclusion, attenuation of field isolates of ASFV remains a promising approach to develop an efficacious and effective vaccine for use in swine production operations. Considerable technical challenges remain to define key correlates of immunity and, in turn, develop robust and consistent manufacturing processes. The optimal immunization protocols and associated safety profiles need to be developed. The potential and observed genetic and phenotypic variability of ASFV make these challenges greater. Very substantial research investments in basic sciences, vaccinology, diagnostics, epidemiology, and clinical disease management are still required.
